# Correction: Multiscale analysis of single and double maternal-zygotic *Myh9* and *Myh10* mutants during mouse preimplantation development

**DOI:** 10.7554/eLife.78088

**Published:** 2022-03-02

**Authors:** Markus Frederik Schliffka, Anna-Francesca Tortorelli, Özge Özgüç, Ludmilla de Plater, Oliver Polzer, Diane Pelzer, Jean-Léon Maître

**Keywords:** Mouse

Schliffka MF, Tortorelli AF, Özgüç O, de Plater L, Polzer O, Pelzer D, Maître J-L. 2021. Multiscale analysis of single and double maternal-zygotic Myh9 and Myh10 mutants during mouse preimplantation development . *eLife* **10**:e68536. doi: 10.7554/eLife.68536

Published 19 April 2021

We have identified an error in the data presented in Fig 2E-H, Fig 5D-E and Appendix table 3.

These data come from the analysis of 10 min long time-lapses that were allegedly acquired with 5 s intervals (see Materials and Methods sections “Microscopy” and “Data analysis - Time-lapse of periodic contractions”).

We have discovered a systemic bug in the acquisition software of our microscope. This resulted in the three initial frames of our time-lapses to be taken every 3 s instead of the requested 5 s steps. The following 118 images would be correctly taken every 5 s. Since the Fourier transform that we have used to characterize periodic movements is sensitive to the framerate and duration of sampling, we have re-analyzed all of the affected data after excluding the three first time points that were acquired with the incorrect delay.

Here, we detail all of the differences that such corrections imply.

Fig 2E: we now show the data over 570 s instead of 600 s. Since we do not remember which vector we had taken originally, we now show a different example vector.

Fig 2F: the power spectra have been updated.

Fig 2G: 4 additional WT embryos show detectable oscillations according to our threshold that is described in the method. None of the mutant condition changes. This affected the p values of the chi squared test. They are lower, in particular they come below 10^–2^ for Myh10 mutants, and therefore solidify our original conclusions.

Fig 2H: the amplitude values have changed, the angle values did not. The correlation is reduced compared to previously (R^2^ goes from 0.406 to 0.370).

Fig 5D: as for 2 F, the power spectra have been updated.

Fig 5E: as for 2 G, 4 additional WT embryos show detectable oscillations. None of the mutant condition changes. This affected the p values of the chi squared test. They are lower and therefore solidify our conclusions.

Appendix table 3: we have changed the values accordingly.

Unrelated to the consequences of the software bug, we found mistakes in 4 instances of the description of our sample size: in the legend of figure 2F, G and H, it should count *15* mzMyh9 embryos instead of *17* as stated; on page 6, it should count *75* instead of *78* when referring to the correlation related to Fig 2H. We have verified that those sample sizes are stated correctly throughout the rest of the paper, including figures and annexes.

We show below the original and corrected versions of Figure 2, Figure 5 and Appendix table 3.

Corrected Figure 2:

**Figure 1:**
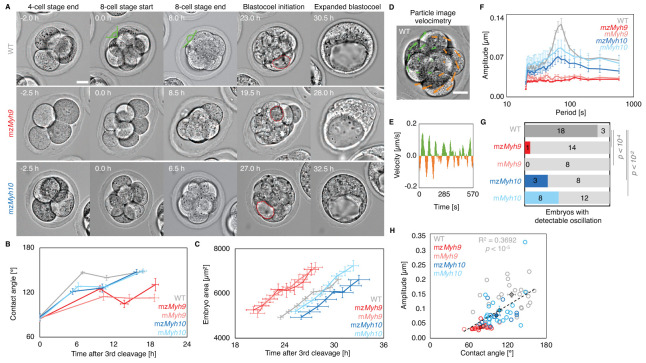


Original Figure 2:

**Figure 2:**
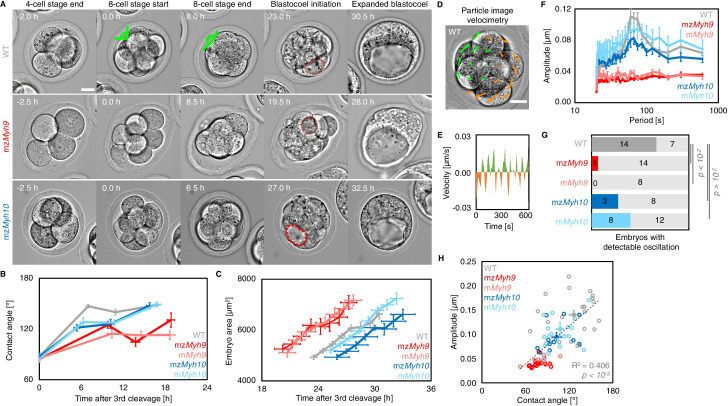


Corrected Figure 5:

**Figure 3:**
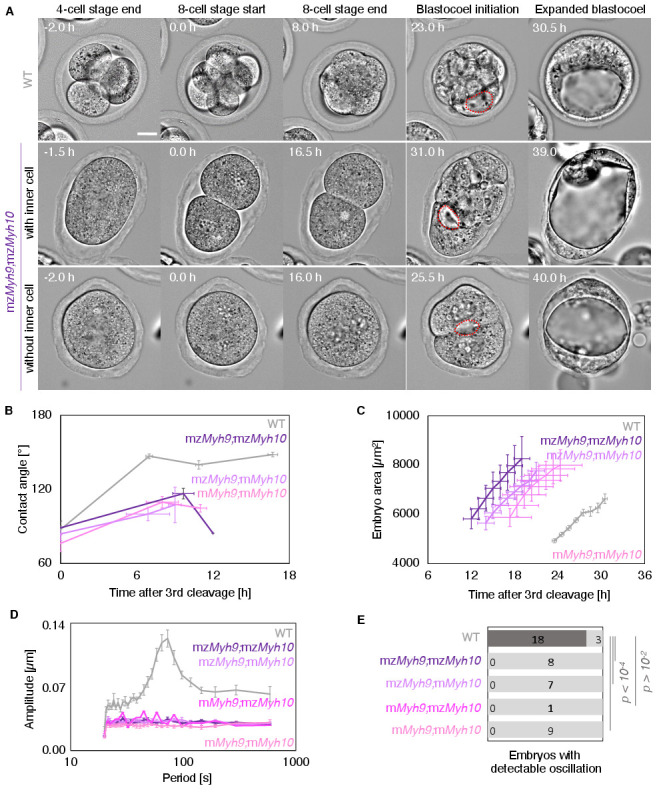


Original Figure 5:

**Figure 4:**
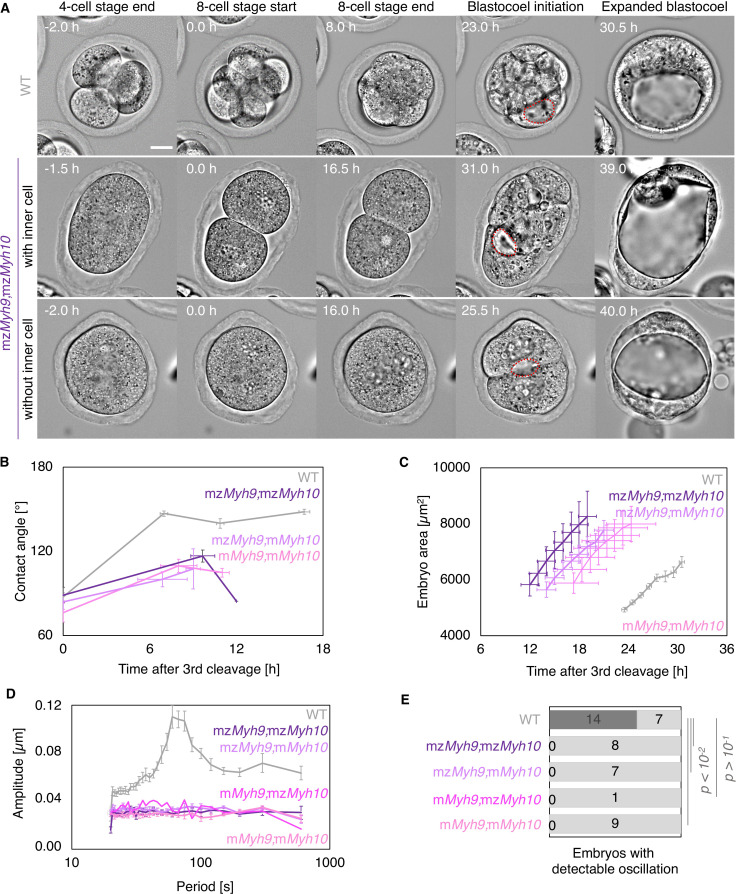


Corrected Appendix table 3:

**Table:** 

		Maximum amplitude within 50–120 s oscillation period range [µm]			Contact angle [°]		
	**embryos**	**mean**	**SEM**	***p* value**	**mean**	**SEM**	***p* value**
WT	21	0.146	0.009	NA	126	5	NA
mzMyh9	15	0.037	0.002	*0.000003*	78	3	*0.000003*
mMyh9	8	0.041	0.003	*0.00002*	82	6	*0.001*
mzMyh10	11	0.077	0.009	*0.004*	103	6	*0.02*
mMyh10	20	0.094	0.014	*0.007*	108	5	*0.02*

Original Appendix table 3:

**Table:** 

		Maximum amplitude within 50–120 s oscillation period range [µm]			Contact angle [°]		
	**embryos**	**mean**	**SEM**	***p* value**	**mean**	**SEM**	***p* value**
WT	21	0.140	0.009	NA	126	5	NA
mzMyh9	15	0.039	0.002	*0.0000005*	78	3	*0.000003*
mMyh9	8	0.045	0.003	*0.00005*	82	6	*0.001*
mzMyh10	11	0.094	0.009	*0.006*	103	6	*0.02*
mMyh10	20	0.111	0.013	*0.01*	108	5	*0.02*

The corresponding sections of the text have been corrected accordingly.

Corrected text:

During the 8 cell stage, contractility becomes visible on the short timescale as periodic contractions, which we can use to gauge the specific contribution of NMHCs (Maître et al., 2015; Maître et al., 2016). We performed particle image velocimetry (PIV) and Fourier analysis to evaluate the period and amplitude of periodic movements (Figure 2D-F) (Maître et al., 2015). While 18/21 WT embryos displayed periodic contractions, these were rarely detected in *Myh9* mutants (1/15 mz*Myh9* and 0/8 m*Myh9* embryos) and occasionally in *Myh10* mutants (3/11 mz*Myh10* and 8/20 m*Myh10* embryos, Figure 2G, Figure 2—video 2). This suggests that contractility is reduced after maternal loss of *Myh10* and greatly reduced following maternal loss of *Myh9*. This hierarchy in the phenotypes of the NMHC paralog mutants parallels the one observed on the long timescale during compaction (Figure 2B). In fact, we find that the amplitude of periodic contractions correlates with the contact angle across the genotypes we considered (Figure 2H, 75 embryos, Pearson’s *R* = 0.608, *P* < 10^–5^, Figure2-Appendix 3). This analysis across timescales reveals the continuum between the short-term impact of *Myh9* or *Myh10* loss onto contractile movements and the long-term morphogenesis, as previously observed for internalizing ICM cells (Maître et al., 2016).

Original text:

During the 8 cell stage, contractility becomes visible on the short timescale as periodic contractions, which we can use to gauge the specific contribution of NMHCs (Maître et al., 2015; Maître et al., 2016). We performed particle image velocimetry (PIV) and Fourier analysis to evaluate the period and amplitude of periodic movements (Figure 2D-F) (Maître et al., 2015). While **14/21** WT embryos displayed periodic contractions, these were rarely detected in *Myh9* mutants (1/15 mz*Myh9* and 0/8 m*Myh9* embryos) and occasionally in *Myh10* mutants (3/11 mz*Myh10* and 8/20 m*Myh10* embryos, Figure 2G, Figure 2—video 2). This suggests that contractility is reduced after maternal loss of *Myh10* and greatly reduced following maternal loss of *Myh9*. This hierarchy in the phenotypes of the NMHC paralog mutants parallels the one observed on the long timescale during compaction (Figure 2B). In fact, we find that the amplitude of periodic contractions correlates with the contact angle across the genotypes we considered (Figure 2H, **78** embryos, Pearson’s *R* = **0.637**, *P* < 10^–5^, Figure2-Appendix 3). This analysis across timescales reveals the continuum between the short-term impact of *Myh9* or *Myh10* loss onto contractile movements and the long-term morphogenesis, as previously observed for internalizing ICM cells (Maître et al., 2016).

Corrected text:

Figure 2: Multiscale analysis of morphogenesis in single maternal-zygotic *Myh9* or *Myh10* mutant embryos.

(A) Representative images of long-term time-lapse of WT, mz*Myh9* and mz*Myh10* embryos at the end of the 4 cell stage, start and end of the 8 cell stage, at the initiation of blastocoel formation and early blastocyst stage (see also Figure 2—video 1). Scale bar, 20 µm. Time in hours after 3^rd^ cleavage. Green lines mark the contact angles increasing during compaction. Dotted red lines indicate the nascent lumen.

(B) Contact angle of WT (grey, n = 23, 23, 21, 22), mz*Myh9* (red, n = 15, 15, 10, 8), m*Myh9* (light red, n = 8, 8, 8, 3), mz*Myh10* (blue, n = 11, 11, 11, 11) and m*Myh10* (light blue, n = 20, 20, 20, 20) embryos after the 3^rd^ cleavage, before and after the 4^th^ cleavage and before the 5^th^ cleavage. Data show mean ± SEM. Statistical analyses are provided in Figure2-Appendix 1–2.

(C) Embryo growth during lumen formation of WT (grey, n = 20), mz*Myh9* (red, n = 9), m*Myh9* (light red, n = 7), mz*Myh10* (blue, n = 7) and m*Myh10* (light blue, n = 13) embryos measured for seven continuous hours after a lumen of at least 20 µm in diameter is observed. Data show mean ± SEM.

(D) Representative image of a short-term time-lapse overlaid with a subset of velocity vectors from Particle Image Velocimetry (PIV) analysis. Green for positive and orange for negative Y-directed movement.

(E) Velocity over time for a representative velocity vector of embryo shown in D and Figure 2—video 2.

(F) Power spectrum resulting from Fourier transform of PIV analysis of WT (grey, n = 21), mz*Myh9* (red, n = 15), m*Myh9* (light red, n = 8), mz*Myh10* (blue, n = 11) and m*Myh10* (light blue, n = 20) embryos. Data show mean ± SEM.

(G) Proportion of WT (grey, n = 21), mz*Myh9* (red, n = 15), m*Myh9* (light red, n = 8), mz*Myh10* (blue, n = 11) and m*Myh10* (light blue, n = 20) embryos showing detectable oscillations in their power spectrum (see Methods). Chi^2^ *p* value comparing to WT is indicated.

(H) Amplitude of oscillation as a function of the mean contact angle for WT (grey, n = 21), mz*Myh9* (red, n = 15), m*Myh9* (light red, n = 8), mz*Myh10* (blue, n = 11) and m*Myh10* (light blue, n = 20) embryos. Open circles show individual embryos and filled circles give mean ± SEM of a given genotype. Pearson’s R^2^ and *p* value are indicated. Statistical analyses are provided in Figure2-Appendix 3.

Original text:

Figure 2: Multiscale analysis of morphogenesis in single maternal-zygotic *Myh9* or *Myh10* mutant embryos.

(A) Representative images of long-term time-lapse of WT, mz*Myh9* and mz*Myh10* embryos at the end of the 4 cell stage, start and end of the 8 cell stage, at the initiation of blastocoel formation and early blastocyst stage (see also Figure 2—video 1). Scale bar, 20 µm. Time in hours after 3^rd^ cleavage. Green lines mark the contact angles increasing during compaction. Dotted red lines indicate the nascent lumen.

(B) Contact angle of WT (grey, n = 23, 23, 21, 22), mz*Myh9* (red, n = 15, 15, 10, 8), m*Myh9* (light red, n = 8, 8, 8, 3), mz*Myh10* (blue, n = 11, 11, 11, 11) and m*Myh10* (light blue, n = 20, 20, 20, 20) embryos after the 3^rd^ cleavage, before and after the 4^th^ cleavage and before the 5^th^ cleavage. Data show mean ± SEM. Statistical analyses are provided in Figure2-Appendix 1–2.

(C) Embryo growth during lumen formation of WT (grey, n = 20), mz*Myh9* (red, n = 9), m*Myh9* (light red, n = 7), mz*Myh10* (blue, n = 7) and m*Myh10* (light blue, n = 13) embryos measured for seven continuous hours after a lumen of at least 20 µm in diameter is observed. Data show mean ± SEM.

(D) Representative image of a short-term time-lapse overlaid with a subset of velocity vectors from Particle Image Velocimetry (PIV) analysis. Green for positive and orange for negative Y-directed movement.

(E) Velocity over time for a representative velocity vector of embryo shown in D and Figure 2—video 2.

(F) Power spectrum resulting from Fourier transform of PIV analysis of WT (grey, n = 21), mz*Myh9* (red, n = **17**), m*Myh9* (light red, n = 8), mz*Myh10* (blue, n = 11) and m*Myh10* (light blue, n = 20) embryos. Data show mean ± SEM.

(G) Proportion of WT (grey, n = 21), mz*Myh9* (red, n = **17**), m*Myh9* (light red, n = 8), mz*Myh10* (blue, n = 11) and m*Myh10* (light blue, n = 20) embryos showing detectable oscillations in their power spectrum (see Methods). Chi^2^ *p* value comparing to WT is indicated.

(H) Amplitude of oscillation as a function of the mean contact angle for WT (grey, n = 21), mz*Myh9* (red, n = **17**), m*Myh9* (light red, n = 8), mz*Myh10* (blue, n = 11) and m*Myh10* (light blue, n = 20) embryos. Open circles show individual embryos and filled circles give mean ± SEM of a given genotype. Pearson’s R^2^ and *p* value are indicated. Statistical analyses are provided in Figure2-Appendix 3.

The article has been corrected accordingly.

